# Cancer du sein au Cameroun, profil histo-épidémiologique: à propos de 3044 cas

**DOI:** 10.11604/pamj.2015.21.242.7269

**Published:** 2015-08-04

**Authors:** Jean Paul Ndamba Engbang, Henri Essome, Valère Mve Koh, Godefroy Simo, Jean Daniel Sime Essam, Albert Sone Mouelle, Jean Louis Oyono Essame

**Affiliations:** 1 Faculté de Médecine et des Sciences Pharmaceutiques de l'Université de Douala, Cameroun; 2 Hôpital Laquintinie de Douala, Cameroun; 3 Centre Médico-Biologique et Cancérologique de Bafoussam, Cameroun; 4 Hôpital Régional de Maroua, Cameroun; 5 Hôpital Général de Douala, Cameroun; 6 Faculté de Médecine et des Sciences Biomédicales de l'Université de Yaoundé I, Cameroun; 7 Centre Hospitalier Universitaire de Yaoundé, Cameroun

**Keywords:** Cancer, sein, épidémiologie, histologie, Cameroun, Cancer, breast, epidemiology, histology, Cameroon

## Abstract

Décrire les caractéristiques épidémiologiques et histo-pathologiques des tumeurs malignes du sein au Cameroun. Il s'agissait d'une étude rétrospective descriptive portant sur les tumeurs malignes du sein, colligées, dans les registres des différents laboratoires d'Anatomie Pathologique publiques et privés repartis dans cinq régions (centre, littoral, Ouest, Nord-ouest, Sud-ouest), pendant une période de 10 ans (2004-2013). Les paramètres étudiés étaient la fréquence, l’âge, le sexe, la localisation, le type et le grade histologique, et les récepteurs hormonaux. Un total de 3044 cas de cancers du sein a été recensé, soit une fréquence annuelle de 304,4 cas en moyenne. Le sexe féminin était le plus représenté avec 2971 cas (97,60%) et les hommes avec 73 cas (2,40%), soit un sexe ratio (H/F) de 0,02. L’âge moyen des patients était de 46±15,87 ans, avec des extrêmes de 13 et 95 ans. Selon la localisation, le sein gauche était atteint dans 1244 cas (52%) et le sein droit dans 1115 cas (47%). Au plan histologique, on retrouvait essentiellement des carcinomes avec 96,50% des cas, des sarcomes 1,39%, des lymphomes 1,07% et la maladie de Paget du mamelon, 1,03%. Les tumeurs épithéliales étaient infiltrantes dans 2049 cas (84,46%), avec une prédominance du carcinome canalaire infiltrant (1870 cas) et non infiltrantes dans 377 cas (15,54%). Le grade histo-pronostic de SBR avait révélé une prédominance du grade II dans 66% des cas. Les cancers du sein restent une pathologie fréquente au Cameroun et atteignent principalement la population féminine en âge de procréer. Ils sont caractérisés par la prédominance du carcinome canalaire infiltrant.

## Introduction

Le cancer du sein est le plus fréquent des cancers féminins dans le monde, il occupe ainsi le premier rang dans la plupart des pays. Il représente 23% des cancers de la femme et 10,9% de tous les cancers humains au monde [1]. En Afrique, Togo a évoqué une variabilité régionale: 16% au Sénégal, 10% en République Sud-Africaine et 4% au Kenya [2]. Au Cameroun, en 1992, le cancer du sein occupait le second rang dans l’étude de Mbakop et al. après celui du col, de la peau et du foie [3]. En 2012 au sein de la population de Yaoundé, le cancer du sein était le cancer le plus fréquent de la femme avec les taux brut d'incidence et standardisé sur l’âge respectivement de 25.89 et 35.25 selon Enow Orock G. et al. [4]. Ces travaux camerounais bien que ne donnant pas la prévalence et l'incidence réelles du cancer du sein au Cameroun, laissent néanmoins constater que cette pathologie connait une forte augmentation ces dernières années (étant passée de la 2e à la 1ere place en 10 ans). Notre étude multicentrique s’était donnée pour objectif le recensement de tous les cas diagnostiqués dans les différents laboratoires agrées d'anatomie pathologie du pays, afin de mieux décrire leurs profils épidémiologiques et histo-pathologiques.

## Méthodes

Il s'agissait d'une étude rétrospective descriptive portant sur les cas de cancers du sein, diagnostiqués sur une période de 10 ans, de 2004 à 2013, dans 12 services et laboratoires d'anatomie-pathologie (publics et privés), répartis dans 5 des 10 régions du Cameroun. Les cas ont été colligés dans les services d'anatomo-pathologie à partir des registres et des dossiers des patients de ces centres de référence de diagnostic des cancers. Le matériel d’étude était constitué de biopsies et des pièces opératoires fixées au formol 10% et traitées selon les règles conventionnelles en anatomopathologie. Les variables étudiées l’âge, le sexe, la localisation de la tumeur, le type et le grade histologique, l'immunohistochimie. L'analyse des variables a été réalisée avec le logiciel Statistical Package for Social Sciences (SPSS), en sa version 16.0.

## Résultats

**Figure 1:**
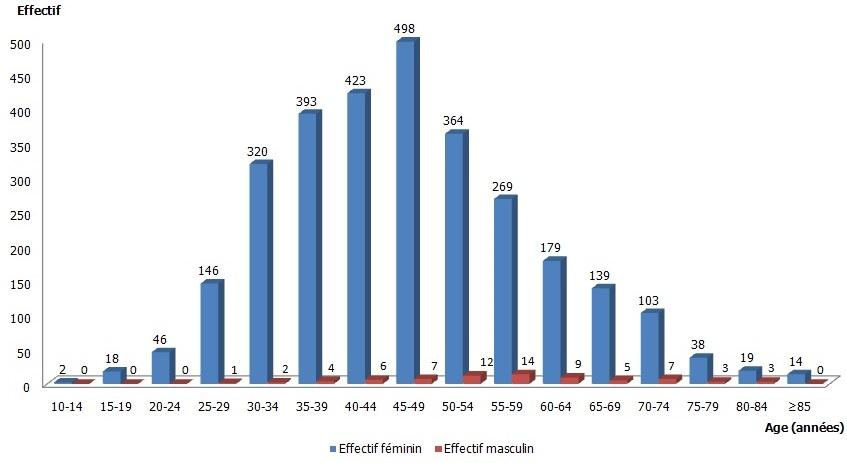
Répartition des patients en fonction du sexe et par tranches d’âge (QSE-quadrant supéro-externe; QSI - quadrant supéro-interne; QII-quadrant inféro-interne; UQ-union des quadrants)

**Aspects épidémiologiques**: durant la période d’étude, l'on a recensé 3044 cas dans l'ensemble des services d'anatomo-pathologie parcourus, ce qui fait une moyenne annuelle de 304,4 par an. Dans l’étude, 2971 cas (97,60%) étaient de sexe féminin et 73 (2,40%) représentaient les hommes, soit un sexe ratio (H/F) de 0,02. L’âge moyen au sein de la population était de 46,80±15,87 ans, avec les extrêmes de 13 et 95 ans (Figure 1). Pour la population féminine, nous avons retrouvé un âge moyen de 46,58±15,72 ans avec des extrêmes variant entre 13 ans et 95 ans. Chez les hommes, la moyenne était établie à 56,00±24,32 ans (extrêmes: 25-83 ans). La localisation a été précisée chez 2394 malades (79,24%). Le coté gauche était le fréquemment concerné avec 52% (n=1244). Le siège par rapport aux quadrants a été rapporté chez 307 patients soit 14,67%. Le QSE (Quadrant Supéro-Externe) représentait 49,51% (Tableau 1).

**Tableau 1:** Distribution des cas selon la localisation

Localisation	Sein droit	Sein gauche	Bilatéral	Total
QSE	70	82	0	152
QSI	20	27	0	47
QII	11	11	0	22
QIE	9	14	0	23
UQ supérieurs	9	18	0	27
UQ inférieurs	4	3	0	7
UQ externes	5	2	0	7
UQ internes	1	2	0	3
Rétromamellonnaire	12	7	0	19
Non précisé	974	1078	35	2087
**Total**	**1115**	**1244**	**35**	**2394**
**Pourcentage (%)**	**47**	**52**	**1**	**100**

P = 0,577; (n = 2394)

**Figure 2:**
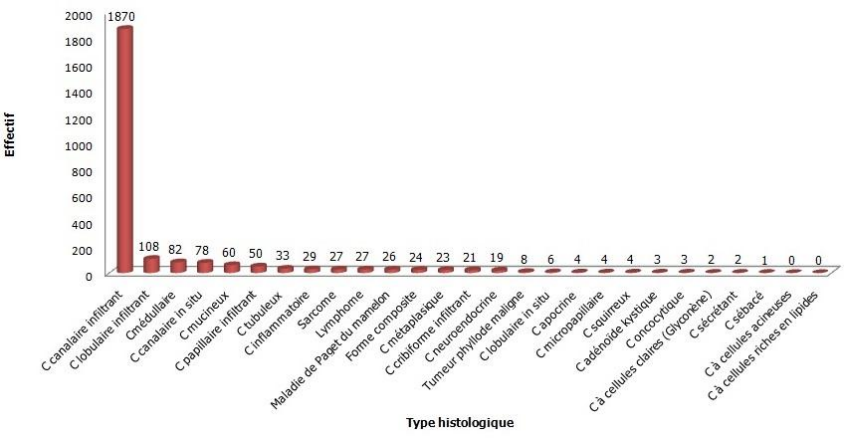
Répartition des cas selon le type histologique (C-Carcinome)

**Aspects histo-pathologiques**: les types histologiques n'ont été précisés que chez 2514 malades (82,58%). Les carcinomes cas ont été les types histologiques les plus fréquents avec 2436 cas (96,50%). Les tumeurs épithéliales étaient infiltrantes dans 2049 cas (84,46%), avec une prédominance du carcinome canalaire infiltrant (1870 cas) (Figure 2). Le grade histologique de Scarff Bloom Richardson (SBR) n'a été précisé que chez 1098 patients (36,07%). Le SBR II a été le plus fréquent avec 66%, suivi du SBR III (20%) et enfin du grade I (14%). Nous n'avons retrouvé les éléments immuno-histochimiques que dans 11 cas. L'immunohistochimie n’étant pratiquée uniquement qu'au Centre Pasteur du Cameroun, et ce depuis 2012. Le RE (récepteur oestrogénique) est positif dans 54,55% des cas et négatif dans 45,45. Par contre, le RP n'est positif que dans moins de 10% des cas.

## Discussion

Nous avons colligé au total 3044 cas de cancers du sein diagnostiqués, soit une fréquence moyenne de 304,4 nouveaux cas par an. Cette fréquence est élevée par rapport à celle trouvée par Zaki et al. au Niger, qui était de 64,5 cas par an [5], Darré et al. au Togo retrouvaient 22,5 cas par an [6]. Dans notre étude, les femmes représentaient 97,60% des cas, soit un sexe ratio (H/F) de 0,02. Valeurs proches de celles retrouvées par Darré T et al. 0,023 [6] et celles de Togo A et al. 0,02 [2]. Ces chiffres sont inférieurs à celles de Zaki et al, 0,03 [5], de même qu’à ceux de Koffi B, 0,03[7]. Ceci pourrait s'expliquer par le fait que ces études étaient majoritairement monocentriques. Concernant l’âge, Les extrêmes étaient 13 et 95 ans, avec comme âge moyen général de 47, 23±12,30 ans. Au sein de la population féminine, l’âge moyen était de 48,13±13 ans et la classe de 45-49 ans était la plus représentée avec 17% des cas. Par ailleurs, 62% des femmes ont un âge inférieur ou égal à 49 ans, correspondant à la période d'activité génitale. Au Niger, Zaki et al retrouvaient 69,89% des cancers du sein chez la femme avant 50 ans [5], valeurs au-dessus des nôtres, et à Dakar, 57,3% de ces tumeurs malignes sont diagnostiqués avant 50 ans selon Dem et al [8], taux un peu inférieur à celui trouvé dans notre série. Toutes ces valeurs sont différentes de celles des autres auteurs dans le monde qui rapportent que 25 à 30% des cas apparaissent avant 50 ans et 55 à 60% entre 50 et 74 ans, âge recommandé pour le diagnostic par Mbra et al. en Cote d'Ivoire [9]. Dans notre étude, la diminution du cancer du sein après 50 ans peut être reliée d'une part au fait que certaines habitudes telles que l'allaitement maternel de durée supérieure à une année sont respectées, d'autre part à l'absence ou à la faible prévalence de certains facteurs de risque tels que les traitements hormonaux, ainsi que la faible consommation d'alcool remarquée au sein de cette population. L'espérance de vie courte également réduit l'apparition du cancer [9]. Toutes ces observations corroborent le caractère hormonal lié au cancer du sein [10].

En revanche, chez les hommes, l’âge moyen était de 52, 20±12,32 ans, la classe d’âge la plus représentée était 55-59 ans avec 19,67% (n=12) et 57,38% des cas ont un âge supérieur ou égal à 55 ans (n=13). Zaki et al. avaient également l'effectif le plus élevé dans la classe 50-60 [5]. Au CHU de Jos, la fréquence la plus élevée était retrouvée entre 60 et 70 ans selon Kidmas et al. [11]. Ces valeurs sont corroborées par l'observation selon laquelle le cancer du sein chez l'homme apparait le plus souvent une à deux décennies plutard que chez la femme [12]. Par ailleurs en Afrique, l'augmentation des maladies hépatiques chez l'homme après 60 ans, entrainant de ce fait une hyperoestrogénie, est un facteur mis en cause dans l'apparition de ce cancer [11]. Sur les 2394 cas précisés, le coté gauche a été le plus atteint avec 52%, suivi du coté droit, 47% et enfin 1% d'atteinte bilatérale. De manière générale, l'atteinte du sein gauche a été prédominante par rapport au sein droit selon plusieurs auteurs [8, 10]. Toutefois, certains comme Darré T et al. et Sano D et al. ont rapporté une prédominance du coté droit [6, 13]. Dans la littérature, l'atteinte bilatérale varie de manière générale entre 3 et 13% [14, 15]. Dans notre étude, la tumeur avait été retrouvée au QSE dans 49,51% des cas, au QSI dans 15,31%, indépendamment du coté du sein atteint. La localisation rétromamellonnaire quant à elle n'a été présente que chez 6,19% des cas. Ces taux sont supérieurs à ceux qu'avaient rapportés Togo A et al. qui ont retrouvé 41,4% pour le QSE, 12,2% pour le QSI; toutefois le siège rétromamellonnaire a été présent chez eux dans 19,05% des cas [2]. Ces écarts peuvent s'expliquer par le fait que les études comparées à cette série sont d'une part clinique, d'autre part les renseignements cliniques fournis par les services d'anatomie pathologie sont parfois incomplets voire même absents pour certains.

Le type histologique a été précisé chez 2514 malades (82,58%). Les cancers infiltrants sont les plus représentés avec 1970 cas (74,38%) pour le carcinome canalaire et 108 cas (4,30%) pour le carcinome lobulaire. La forme composite, constituée de deux à plusieurs carcinomes différents en proportions variables, est présente dans 24 cas (0,95%). Le carcinome médullaire arrive au cinquième rang avec 1,88% des cas. Darré et al. au Togo ont retrouvé, tout comme dans notre série, les mêmes types histologiques mais dans des proportions différentes à savoir: carcinomes (96%), sarcomes (2,6%), lymphomes (1,33%) [6], alors que Echimane et al. rapportaient seulement deux groupes, les carcinomes (97,16%) et les sarcomes (2,04%) [16]. Le grade histopronostic de SBR étudié dans cette série a révélé la prédominance du grade II de l'ordre de 60%, suivi du grade III à 20%. Ce classement a également été celui de Darré et al. au Togo mais avec des proportions différentes à savoir II (54,67%), III (34,95%) et I (10, 38%) [6]. A l'Hôpital Gynéco-obstétrique et pédiatrique de Yaoundé, Essiben et al. retrouvaient une tendance différente, avec dans l'ordre de fréquence, les grades II, I et III [17]. A Bangui, le grade II a été le plus en vue avec 58,4%, alors que les grades I et II étaient dans les mêmes proportions (20,8%), selon Koffi et al.[7]. Ces observations sont contraires à celles rapportées à Libreville par Meye et al. selon lesquelles, le grade II occupait la dernière position après les grades III et I [18]. Le statut immunohistochimique a été très peu étudié, seulement 11 cas rapportés (0,36%). Cette proportion était petite par rapport à toute la population étudiée et ne saurait traduire de manière fiable la réalité. Ceci s'explique par l'introduction récente de ce type d'analyse dans le paquet diagnostic du pays et uniquement présent au centre Pasteur du Cameroun pour l'instant, et dont l'accès reste restreint pour des raisons pécuniaires. Pourtant, le rôle pronostique et prédictif des récepteurs hormonaux reste indispensable dans la prise en du cancer du sein [19]. Néanmoins, malgré le petit effectif, le taux trouvé de 54,54% de RE+, n'est pas très éloigné des ceux trouvés par Togo (58, 10%) [2], toutefois reste plus bas que la valeur qu'avait révélée Blanchard (78,2%) [20].

## Conclusion

Les cancers du sein restent une pathologie fréquente au Cameroun et sont caractérisés par une atteinte beaucoup plus importante du sexe feminine, principalement la population jeune (en âge de procréer) et par la prédominance du carcinome canalaire infiltrant.
